# Analysis of In Silico Properties and In Vitro Immunomodulatory Effects of Seven Synthetic Host Defence Peptides in Gilthead Seabream (*Sparus aurata*) Leucocytes

**DOI:** 10.1007/s10126-025-10488-z

**Published:** 2025-07-12

**Authors:** Claudia Marín-Parra, Jhon Alberto Serna-Duque, Cristóbal Espinosa-Ruiz, María Ángeles Esteban

**Affiliations:** 1 Immunobiology for Aquaculture Group, Department of Cell Biology and Histology, Faculty of Biology, Campus Regional de Excelencia Internacional “Campus Mare Nostrum”, University of Murcia, 30100 Murcia, Spain; 2 Aquaculture and Animal Production Technology Department, Institute of Agricultural and Environmental Research and Development of Murcia (IMIDA), 30150 Murcia, Spain

**Keywords:** Host defense peptides** (**HDPs), Piscidins, Hepcidins, In silico analysis, Gene expression, *Sparus aurata*

## Abstract

Host defense peptides (HDPs) are vital to immunity due to their antimicrobial and immunomodulatory properties. While extensively studied in mammals, their immunomodulatory roles remain complex, and research on HDPs in fish is limited. This study aimed to predict physicochemical properties and mechanisms of action of seven HDPs in gilthead seabream (*Sparus aurata*): two piscidins (piscidin 1, piscidin 2) and five hepcidins (hepcidin H1, H2C, H2E, H2H, H2I). Predictions indicated heterogeneity among HDPs, all exhibiting antioxidant capacity and bioactive potential. In vitro analyses of these synthetic HDPs on head kidney leucocytes of gilthead seabream revealed minimal direct effects on leucocyte activities. RT-PCR gene expression analysis in leukocytes after 2 h of HDP incubation showed significant upregulation of *bax-1* (hepcidin H2I), *il-6* (piscidin 2), *tnf-α* (piscidin 1), *tlr-7* (piscidin 1), and *tlr-8* (piscidin 1), and downregulation of *casp-3* (hepcidin H1), *bcl-2* (hepcidin H2C), *il-1β* (hepcidin H1, H2C, H2E, H2H, H2I), *tnf-α* (piscidin 2, hepcidin H1, H2E, H2I), *tlr-7* (piscidin 2, hepcidin H2E), and *tlr-8* (piscidin 2, hepcidin H2H). The results indicate that HDPs demonstrate diverse immunomodulatory impacts on seabream white blood cells, playing a crucial role in regulating genes associated with programmed cell death, inflammatory responses, and Toll-like receptors. This complex function highlights the adaptability and significance of the studied HDPs in the immune system of gilthead seabream.

## Introduction

Antimicrobial peptides (AMPs) are crucial components of the innate immune system in many organisms (Afacan et al. 2012). To date, more than 3500 natural AMPs have been identified and, although they are mainly found in eukaryotic organisms, they have also been found in prokaryotes (Wang et al. 2015). They are amphiphilic compounds characterised by short amino acid sequences (usually between 12 and 50 amino acids) and an overall cationic charge (Roca-Pinilla et al. 2022). Early studies on AMPs focused on corroborating their direct biocidal activity, as they kill or inhibit the growth of bacteria, parasites, viruses and fungi (Lazzaro et al. 2020; Nguyen et al. 2011). In addition, a wide range of immunomodulatory functions associated with AMPs have become apparent in recent decades (Nijnik and Hancock 2009), suggesting further indirect capabilities of AMPs to combat pathogens through modulation of the immune system, i.e. biocidal activity by indirect methods (Katzenback 2015). This has led to the term host defence peptides (HDPs) being preferred to AMPs, as it better reflects the fact that they possess both antimicrobial activity and immunomodulatory properties.

The most studied HDPs in vertebrates are defensins and cathelicidins, which possess chemotactic and angiogenic activity, modulate cytokine expression, reactive oxygen species (ROS) and nitrogen species, induce immune cell differentiation and enhance wound healing (Bin Hafeez et al. 2021). HDPs studied in fish include piscidins, hepcidins, defensins, cathelicidins and histone-derived peptides. Piscidins are a family of HDPs unique to teleosts that have been characterised in species of the superorder Acanthopterygii. Belonging to this piscidin family are piscidin itself, moronecidin, pleurocidin, epinecidin, gaduscidin, misgurin, dicentrazine, chrysophysin and myxinidin (Fernandes et al. 2010). Piscidins were first identified in hybrid striped bass (*Morone chrysops* × *M.saxatilis*) mast cells (Silphaduang and Noga 2001; Bhat et al. 2022). Piscidins are linear cationic cationic α-helical peptides with a potent and broad-spectrum demonstrated antimicrobial activity against bacteria and ectoparasites (Noga et al. 2009; Colorni et al. 2008). The functions of piscidins have recently been reviewed (Zaccone et al. 2022; Asensio-Calavia et al. 2023).

On the other hand, hepcidin is a cysteine-rich peptide first discovered in humans but subsequently also identified in numerous vertebrate species (Jin et al. 2023). Most mammals (with the exception of the mouse) have only one hepcidin gene (*hamp1*) that serves a dual function by regulating iron metabolism and responding to antimicrobial agents (Hilton and Lambert 2008). However, many teleost fish have multiple copies of the hepcidin gene. This is probably due to known genome duplications in these animals and positive Darwinian selection. In addition, it has been suggested that different copies of hepcidin in fish may have different functions (Padhi and Verghese 2007). In fish, hepcidin was first also identified and studied in the hybrid striped bass, although it is the synthetic hepcidin TH1-5 from tilapia (*Oreochromis niloticus*) that has been most studied in teleosts (Shike et al. 2002; Gui et al. 2016). Some fish contain clusters of two or more copies of hepcidin (Meyer and Van de Peer 2005; Boumaiza and Abidi 2019). The available results show that it is widely accepted that teleost fish have two types of hepcidin that have different functions. While hepcidin 1 (*hamp1*) is more involved in the regulation of iron metabolism, hepcidin 2 (*hamp2*) plays a key role in antimicrobial capacity (Shi and Camus 2006; Neves et al. 2015). In fact, two types of hepcidins have been identified in many fish species including Japanese flounder (*Paralichthys olivaceus*) (Hirono et al. 2005)), largemouth bass (*Micropterus salmoides*) (Robertson et al. 2009), orange-spotted grouper (*Epinephelus coioides*) (Zhou et al. 2011), turbot (*Scophthalmus maximus*) (Pereiro et al. 2012), spotted scat (*Scatophagus argus*) (Gui et al. 2016), and pufferfish (*Takifugu pardalis*) (Go et al. 2019). However, three different hepcidins were described in Mozambique tilapia (*Oreochromis mossambicus*) (Hsieh et al. 2010; Huang et al. 2007). In addition, fish hepcidins have been shown to be up-regulated in response to pathogens and appear to have a wide range of direct antibacterial activity against a variety of pathogens. There are also some studies showing hepcidins induced following exposure to organic pollutants such as benzo(a)pyrene (Wang et al. 2009; Chen et al. 2008).

Gilthead seabream (*S. aurata*) is a fish species of great importance in the Mediterranean aquaculture. In recent years, our research team has carried out extensive studies that have led to the identification of around 30 seabreams derived HDPs. Bioinformatic analyses were used to characterize for the first time in this species two paralogous piscidin genes, one type I hepcidin and fourteen type II hepcidins (Serna-Duque et al. 2022a). Hepcidin transcription profiles in response to bacterial challenges differed based on the type of hepcidin and tissue, indicating different immune roles. Additionally, hepcidin genes were also associated with iron homeostasis, storage, and regulation in this species (Serna-Duque et al. 2022c). Besides this, in the early infection stages of *Vibrio harveyi*, the expression of genes encoding hepcidins and piscidins was evaluated. The results demonstrated varying expression levels depending on the type of HDPs and the tissue examined. Curiously, while hepcidin played a role in all the tissues studied (skin, mucus, blood, skin, head, kidney, liver and spleen), the piscidins were restricted to the spleen (Serna-Duque et al. 2023). More recently, different hepcidin expression patterns were also analysed in seabream that received λ-carrageenan injections to induce a sterile acute inflammatory response (Campos-Sánchez et al. 2024). Although there are several lines of evidence that support the involvement of these HDPs in the modulation of the immunity of the sea bream in vivo, it is still unknown whether they have a direct effect on the immune cells. Therefore, the aim of the present study was to investigate the direct effects of seven synthetic HDPs on the viability of seabream leucocytes and on their primary activities. Furthermore, their effect on modulating various genes involved in apoptosis, inflammation and Toll-like receptors was also evaluated. By correlating HDP gene expression with established cellular immune functions, our functional approach advances from descriptive transcriptomics to a comprehensive understanding of immune functionality. This methodology assesses the impact of HDP regulation on pathogen defense, immune modulation, and inflammation control within a specific context. It identifies HDPs as biomarkers and mediators of immune function, thereby substantiating their central role in fish defense.

## Materials and Methods

### Host-Defence Peptides

Freeze-dried synthetic peptides from gilthead seabream were purchased (GenScript), dissolved in Milli-Q® water (pH: 6.988) and L-15 culture medium (Leibovitz) was used to adjust peptide desired concentrations. They were stored at −20 °C. The HDPs were: two piscidins (1 and 2) and five hepcidins (H1, H2C, H2E, H2H and H2I). The sequences of the used HDPs are presented in Table 1.

**Table 1:** Sequences of synthetic host defense peptides (HDPs) from gilthead seabream (*Sparus aurata*)

HDPs	Sequences	Number of amino acids
Piscidin 1	FIGWLIKGAISAGKAIHGAIRRRFG	25
Piscidin 2	FIGLLISGAITAGTMIH	17
Hepcidin H1	QSHISMCYYCCNCCRANKGCGYCCK	25
Hepcidin H2C	CRFCCRCCPRMRGCGLCCRF	20
Hepcidin H2E	CRFCCGCCPNMIGCGTCCKF	20
Hepcidin H2H	SPAGCKFCCGCCPNMRGCGVCCRF	24
Hepcidin H2I	SPADCEFCCGCCPDMTGCGICCRF	23

### Prediction of Physicochemical Properties and Bioactivity of the Peptides

The secondary structures of the HDPs were predicted using ChimeraX 1.8, with the AlphaFold tool, which employs a deep learning algorithm implemented in AlphaFold2 through Google Colab (https://colab.research.google.com/github/sokrypton/ColabFold/ (accessed on 19 May 2024)) (Jumper et al. 2021). In silico analysis were carried out to predict the physicochemical properties and the potential bioactivity of the selected HDPs. ADP (https://aps.unmc.edu/) was used to predict hydrophobic ratio, net charge, molecular weight, and Boman index. ProtParam in ExPASy (https://web.expasy.org/protparam/) was used to calculate the instability and aliphatic index (Gasteiger et al. 2005). Instability index values below 40 indicated HDPs stability (Guruprasad et al. 1990). The antimicrobial activity was predicted using the Artificial Neural Network (ANN) classifier implemented in “CAMPr3” (Waghu et al. 2015). To determine whether the HDPs could function as antiviral peptides, a deep learning-based framework was utilized “AVP-IFT” (https://awi.cuhk.edu.cn/∼dbAMP/AVP/) (Guan et al. 2024). The antifungal activity was predicted using a binary model “Antifp” (http://webs.iiitd.edu.in/raghava/antifp) (Agrawal et al. 2018). The anti-parasitic activity was predicted using the webpage “AMPfun” (http://fdblab.csie.ncu.edu.tw/AMPfun/index.html) (Chung et al. 2019). The bioactivity was predicted by Peptide Ranker (http://distilldeep.ucd.ie/PeptideRanker/). Peptide sequences are considered likely to be bioactive if the prediction is over 0.8 (Mooney et al. 2012). To predict the potential biological activities of the HDPs. BIOPEP-UWM (https://biochemia.uwm.edu.pl/biopep/start_biopep.php) database of bioactive peptides (currently includes 73 activities) was used (Minkiewicz et al. 2019). The potential biological activities of the sequences were calculated using the parameter A, which indicates the frequency of occurrence of fragments in a sequence showing a specific activity that was selected (Dziuba et al. 2022).

### Fish

A total of 35 juvenile gilthead seabreams *(Sparus aurata)* with an average weight of 31 g ± 8 and a length of 13 cm ± 3, were obtained from a local farm (Alicante, Spain). Prior to the sampling the fish were subjected to a 15-day quarantine period in a seawater recirculating system (500 L, 28 ‰ salinity and 22 ºC ± 2 ºC water temperature). The artificial photoperiod was established at 12 h light and 12 h darkness. Partial water changes were performed daily, and fish were fed 1% of their body weight on a commercial pelleted diet [Skretting, Spain, consisted of crude protein (48.5%), crude oils and fats (18%), crude ash (6.8%), crude fiber (2.2%), phosphorus (0.9%), calcium (0.9%), and sodium (0.3%)].

### Head-Kidney Leucocytes Isolation and Incubation with HDPs

**Fig. 1:**
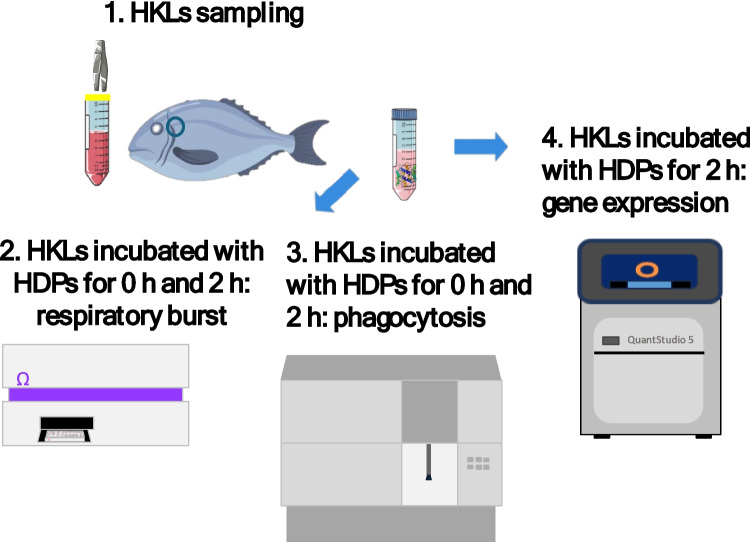
Schematic representation of the study's experimental design

Fish were euthanized using clove oil (Guinama) and rapidly bleeding. Isolation of head-kidney leucocytes (HKLs) were done following a previous described protocol (Esteban et al. 1998). Head-kidney was removed and forced to pass through cell filters (100 μm of pore size, Fisherbrand™) with 20 mL of L-15 medium (Leibovitz) (Fig. 1). The culture media was previously supplemented with 0.35% sodium chloride (to adjust the medium osmolality to gilthead seabream’s plasma osmolarity), 1% heparin (Biowest, to avoid coagulation), penicillin (Biowest, to avoid contamination), L-glutamine (Biowest) and 3% Fetal Calf Serum (FCS, Gibco). Cells were centrifugated (485 × g, 10 min), resuspended and washed twice using L-15 supplemented medium without heparin. Cell concentration was determined by an automatic counting chamber (BioRad) and adjusted to 10^7^ cells mL^−1^.

The viability of HKLs was evaluated by flow cytometry using propidium iodide (PI, a red fluorescent dye widely utilised to identify non-viable cells). Aliquots of 50 μL of PI solution (400 mg mL^−1^, Sigma-Aldrich) were added to 5 mL Falcon tubes containing 100 μL of HKLs (from 5 different fish analysed independently, n = 5) previously incubated for 0 h or 2 h with HDPs (at final concentrations of 0 µM (control), 12.5 µM and 25 µM). Before analysis, the samples were gently mixed. Flow cytometry analysis was done on a FACScan flow cytometer (Becton Dickinson) equipped with a 488 nm argon-ion laser and 10,000 cells were recorded per sample at a rate of 300 cells s^−1^. Data were acquired by two-parameter side scatter (SSC) and forward scatter (FSC). Graphical representations in the form of dot plots or histograms were generated for red fluorescence (FL2). For positive controls, 50 μL of trypan blue were added to lyse cells. Viable cells were calculated by resting 100% to the estimated dead cells, which are the percentage of cells displaying PI (red) fluorescence.

### Immune Parameters

Respiratory burst activity of HKLs (in 5 fish) was determined using a chemiluminescence method (Bayne and Levy 1991). HKLs (100 µL well^−1^) were dispensed into flat-bottomed 96-well microtiter plates (Nunc) and incubated for 0 h or 2 h with the selected HDPs at the final concentrations indicated above. Then, 100 µL of HBSS (Hank’s balanced salt solution, Gibco) containing 1 μg mL^−1^ phorbol myristate acetate (PMA, Sigma) and 10^−4^ M luminol (Sigma) were added to each well. Immediately, luminescence from cells was detected using a FLUOstar Omega Microplate Reade (BMG LABTECH) for 1 h at 2 min intervals. Kinetics data were analysed, and maximum ROS production (slope min^−1^) was calculated.

Flow cytometry was also used to assess the effect of HDPs on the phagocytic activity of HKLs (Rodríguez et al. 2003a). Briefly, yeast cells (*Saccharomyces cerevisiae*; strain S288C) were adjusted to 10^8^ yeast cells mL^−1^ before being labelled with fluorescein isothiocyanate (FITC Sigma-Aldrich) and heat-inactivated (30 min at 60 ºC) (Rodríguez et al. 2003b). Samples of 100 μL of HKLs previously incubated with HDPs were placed into 5 mL glass tubes (Falcon, Becton–Dickinson) and aliquots of 60 μL of FITC-labelled yeast cells were added to each tube. Samples were incubated (dark, 30 min, 22 °C) and afterwards, the mix were resuspended and centrifuged (400 × *g,* 5 min). To stop phagocytosis, the samples were placed on ice before adding 50 μL of ice-cold trypan blue solution (0.5% in PBS) to each one, to quench the fluorescence of extracellular yeast cells. Samples were analysed by a FACScan flow cytometer which was configured to analyse the phagocytic cells, characterized by having the highest side scatter (SSC) and forward scatter (FSC) values. The percentage (%) of HKLs with ingested yeast cells was defined as phagocytic ability and phagocytic capacity (expressed as arbitrary units, a.u.) was establish as an estimation of the proportion of yeast ingested by each phagocyte.

### Gene Expression

To assess gene expression (in three fish), samples of 7.5 × 10^4^ HKLs previously incubated for 2 h with HDPs, were centrifuged (400 × *g*, 10 min, 22 ºC) and the supernatants were removed. The pelleted cells were stored at – 80 ºC for gene expression analysis. Total RNA was extracted using the PureLink® RNA Mini Kit (Life Technologies) according to the manufacturer´s guidelines. The Nanodrop® spectrophotometer was used to determine the total RNA concentration and assess its purity. RNA samples were stored at – 20 ºC until used. Then, 1 µg of RNA underwent DNase I treatment (Invitrogen) to eliminate genomic DNA contamination. Subsequently, complementary DNA (cDNA) was synthesized using SuperScript IV reverse transcriptase (Invitrogen) with an oligo-dT18 primer. Gene expression was studied by a Fast real-time qPCR using a QuantStudio™ 5 Real-Time PCR System (Applied Biosystems). The 10 µM reaction mixture contained: 5 μL of SYBR Green supermix, 2.5 μL of the selected primers (10 µM, working solution) (Table 2) and 2.5 μL of cDNA. The reaction mixtures were first incubated for 10 min at 95 °C, followed by 40 cycles of 15 s at 95 °C, 1 min at 60 °C, and finally, 15 s at 95 °C, 1 min at 60 °C, and 15 s at 95 °C. The gene expression results were determined using the comparative cycle threshold (Ct) method (Livak and Schmittgen 2001). The reference genes chosen for the normalisation were translation elongation factor (*ef1a*) and ribosomal protein S18 (*rps18*) (Table 2) (Cordero et al. 2016). Genes selected for the study were apoptosis-related genes *casp-3, bax-1* and *bcl-2* (Morcillo et al. 2016)*,* genes involved in inflammation *il-1β*, *il-6* (Cordero et al. 2016),* tnf-α* (Campos-Sánchez et al. 2021)*, tgfβ*, *il-10* (Cordero et al. 2016) and toll-like receptors (TLR), *tlr-5, tlr-7* and *tlr-8* (Campos-Sánchez et al. 2021).

**Table 2:** Primer sequences used for real-time qPCR analysis of gene expression in gilthead seabream (*Sparus aurata*)

Target gene	ID primer	Primer sequences (5′- 3′)	Accession number	References
Translation elongation factor	*ef1a*	F: TGTCATCAAGGCTGTTGAGC R: GCACACTTCTTGTTGCTGGA	AF184170	[47]
18S ribosomal RNA	*rps18*	F: CGAAAGCATTTGCCAAGAAT R: AGTTGGCACCGTTTATGGTC	AM490061	[47]
Caspase-3 (casp-3)	*casp-3*	F: CTGATCTGGATGGAGGCATT R: AGTAGTAGCCTGGGGCTGTG	EU722334	[47]
Bcl-2-associated protein X	*bax-1*	F: CAACAAGATGGCATCACACC R:TGAACCCGCTCGTATATGAAA	AM963390	[48]
B-cell lymphoma 2	*bcl-2*	F: TCAGGAGTGATGTCGAGCTG R: CAGCCAGGTGCTGACATAGA	FM145663	[48]
Interleukin-1b	*il-1β*	F: GCGAGCAGAGGCACTTAGTC R: GGTAGGTCGCCATGTTCAGT	AJ277166	[47]
Interleukin-6	*il-6*	F: AGGCAGGAGTTTGAAGCTGA R: ATGCTGAAGTTGGTGGAAGG	AM749958	[47]
Tumor necrosis factor	*tnf-α*	F: CTGTGGAGGGAAGAATCGAG R: TCCACTCCACCTGGTCTTTC	AJ413189	[47]
Interleukin-10	*il-10*	F: CTCACATGCAGTCCATCCAG R: TGTGATGTCAAACGGTTGCT	FG261948	[47]
Transforming growth factor	*tgf- 1β*	F: GCATGTGGCAGAGATGAAGA R: TTCAGCATGATACGGCAGAG	AF424703	[47]
Toll-like receptor 5	*tlr5*	F: CAACTTGAGCTCCAACGCAC R: GGCTGGAGATAGGTCAAGGC	B001824	[49]
Toll-like receptor 7	*tlr7*	F: CCAACAATGGGAGCATGGTG R: ATGGTGAGAGTCAGGTTG GTG	B004477	[49]
Toll-like receptor 8	*tlr8*	F: CCAGAGCAATTCCAGGGCTA R: TGTCCAGCCCTTTGAACTCTG	B024796	[49]

### Statistical Analysis

All data are presented as mean ± standard error of the mean (SEM). SPSS (25.0 version; SPSS Inc., Chicago, IL, USA) for MAC was used to perform statistical evaluation. One-way analysis of variance (ANOVA) followed by Tukey (parametric) or Games-Howell (non-parametric) tests were used to detect differences due to incubation of HKLs with HDPs. If possible, data was transformed for data normality. The level of significance was set at *p* < 0.05. Correlations between physicochemical properties and immune parameters were analysed. The correlation matrix was generated utilizing GraphPad Prism software (version 10.0.0, GraphPad Software, Boston, MA, USA). Positive correlation was considered at > 0.5.

## Results

### In Silico* Predictions*

**Fig. 2:**
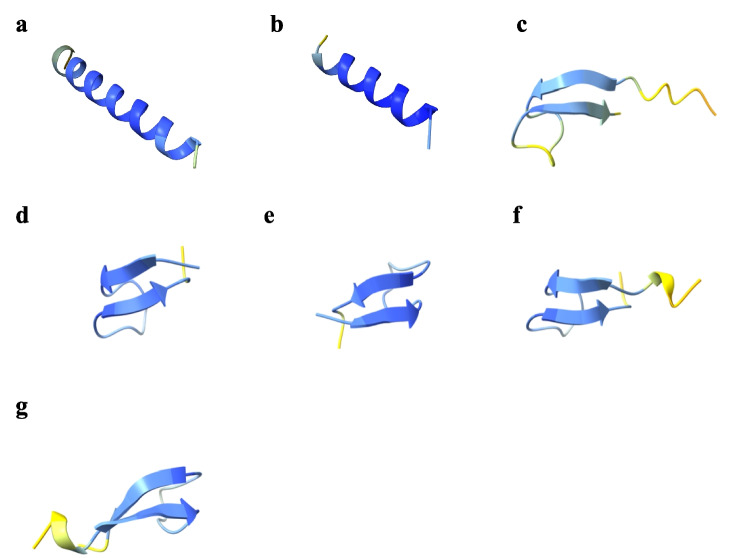
Secondary structure of: **a**) piscidin 1, **b**) piscidin 2, **c**) hepcidin H1, **d**) hepcidin H2C, **e**) hepcidin H2E, **f**) hepcidin H2H and **g**) hepcidin H2I predicted by AlphaFold2 using ChimeraX 1.8 software

Piscidin 1 and piscidin 2 predominantly adopt an α-helical conformation (Fig. 2), with most of the structure predicted with high confidence (> 90%). However, the terminal regions of the helices were predicted with slightly lower confidence (70–90%). In contrast, hepcidins adopt a β-sheet conformation (Fig. 2), with most of their structure also predicted with moderate to high confidence (70–90%).

The physicochemical properties of HDPs were predicted in silico, and the results are summarized in Table 3. In terms of hydrophobicity, piscidin 2 has a higher hydrophobicity index (59%) than piscidin 1 (52%). The hydrophobic ratio of the hepcidins varied between 44% (hepcidin H1) and 60% (hepcidin H2C and H2E). Piscidin 1 displayed the highest net positive charge among the selected peptides, while piscidin 2 had a net charge of 0.25. Hepcidin H2I had a negative net charge of −2, while the other hepcidins had net charges ranging from 2 (hepcidin H2E) to 5 (hepcidin H2C). The aliphatic index of HDPs ranged from 16.3 (hepcidin H2H) to 149.4 (piscidin 2). The MW of the HDPs ranged from 1715.094 (piscidin 2) to 2842.41 (hepcidin H1). The Boman index (potential protein interaction) for piscidin 1 and 2 was 0.56 and −1.65, respectively, while that for hepcidins ranged from 0.16 (hepcidin H2E) to 2.46 (hepcidin H2C). The grand average hydropathicity index (GRAVY) represents the average hydropathicity of the peptide. Piscidin 1 and 2 had GRAVY index values of 0.44 and 1.61, respectively, while hepcidin GRAVY values ranged from −0.16 (hepcidin H1) to 0.83 (hepcidin H2E) (Table 3). The results of the instability index showed that the majority of HDPs were stable, with piscidin 2 and hepcidin H2C being the exceptions (instability index > 40). Piscidin 1 and piscidin 2 had the highest aliphatic index (109.6 and 149.4, respectively) among the studied HDPs, while the index for hepcidins ranged from 16.25 for hepcidin H2H to 20.42 for hepcidin H2I (Table 3). In silico predictions indicate that all HDPs may have antimicrobial activity (Table 4)*. *In silico analyses suggest that Hepcidin H1 possesses potential antiviral, antifungal, and antiparasitic activities. Piscidin 2 appears to exhibit antiviral and antiparasitic activity, while Hepcidin H2C may demonstrate antiviral and antifungal properties. Additionally, Piscidin 1, Hepcidin H2E, and Hepcidin H2H are predicted to have antifungal activity, whereas Hepcidin H2I shows potential antiviral activity. The peptide ranking score was used to predict the likelihood of HDPs being bioactive, with most peptides falling between 0.96 and 1.00, except for piscidin 2, which scored 0.46 (Table 5). According to the report, all piscidins and four hepcidins were predicted to have ACE inhibitory activity, whereas piscidin 1 and all hepcidins were predicted to have antioxidative activity. Besides this, Biopep predicted that all HDPs had dipeptidyl peptidase III and IV inhibitory activity. CaMPDE and renin inhibitory activities were reported for piscidin 1, hepcidin H2E, hepcidin H2H, and hepcidin H2I, whereas only piscidins showed stimulating activity. Furthermore, hepcidin H1 was predicted to have activating ubiquitin-mediated proteolysis activity, and hepcidin H2I was predicted to have hypolipidemic activity (Table 6).

**Table 3:** Physicochemical properties of the host defense peptides (HDPs) predicted in silico. Parameters include: hydrophobic ratio (percentage of hydrophobic amino acids), net charge (overall electric charge at pH 7), MW (molecular weight), Boman index (binding potential to proteins), GRAVY (grand average of hydropathy), instability index (≥ 40 indicates an unstable peptide), and aliphatic index (indicator of thermostability)

HDPs	Hydrophobic ratio (%)	Net charge	MW (da)	Boman index (kcal/mol)	Gravy	Instability index	Aliphatic index
Piscidin 1	52%	5.25	2696.24	0.56	0.44	49.1	109.6
Piscidin 2	59%	0.25	1715.09	−1.65	1.61	6.68	149.41
Hepcidin H1	44%	3.25	2842.41	1.42	−0.16	51.22	19.6
Hepcidin H2C	60%	5	2374.99	2.46	0.32	7.35	19.5
Hepcidin H2E	60%	2	2149.73	0.16	0.83	14.89	19.5
Hepcidin H2H	54%	3	2503.11	0.71	0.48	37.26	16.25
Hepcidin H2I	54%	−2	2522.02	0.71	0.54	29.99	20.42

**Table 4:** In silico prediction of antimicrobial (AMP), antiviral (ANV), antifungal (ANF), antiparasitic (ANP) activities using CAMPR3, AVP-IFT, Antifp, and AMPFUN tools. Predicted inactivity is labelled as NA (no predicted activity)

HDPs	Antimicrobial	Antiviral	Antifungal	Antiparasitic
Piscidin 1	AMP	NA	ANF	NA
Piscidin 2	AMP	ANV	NA	ANP
Hepcidin H1	AMP	ANV	ANF	ANP
Hepcidin H2C	AMP	ANV	ANF	NA
Hepcidin H2E	AMP	NA	ANF	NA
Hepcidin H2H	AMP	NA	ANF	NA
Hepcidin H2I	AMP	ANV	NA	NA

**Table 5:** Summary of the main potential activities of the host defense peptides (HDPs) determined by using Peptide Ranker (bioactive > 0.8) and BIOPEP UWM. Predicted inactivity is labelled as NA (no predicted activity)

HDPs	Bioactive potential	Cell penetration potential	ACE inhibitor	Antioxidative	CaMPDE inhibitor	Dipeptidyl peptidase III inhibitor
Piscidin 1	0.96	0.23	0.76	0.08	0.04	0.2
Piscidin 2	0.47	0.08	0.41	NA	NA	0.06
Hepcidin H1	1	0.09	NA	0.08	NA	0.08
Hepcidin H2C	0.99	0.39	0.25	0.05	NA	0.2
Hepcidin H2E	0.99	0.09	0.2	0.05	0.05	0.05
Hepcidin H2H	1	0.12	0.21	0.04	0.04	0.08
Hepcidin H2I	1	0.06	0.21	0.08	0.04	0.04

**Table 6:** Continuation of the summary of the main potential activities of the host defense peptides (HDPs) determined by using BIOPEP UWM. Predicted inactivity is labelled as NA (no predicted activity)

HDPs	Dipeptidyl peptidase IV inhibitor	Renin inhibitor	Stimulating	Activating ubiquitin-mediated proteolysis	Hypolipidemic
Piscidin 1	0.48	0.04	0.04	NA	NA
Piscidin 2	0.59	NA	0.12	NA	NA
Hepcidin H1	0.28	NA	NA	0.04	NA
Hepcidin H2C	0.2	NA	NA	NA	NA
Hepcidin H2E	0.2	0.05	NA	NA	NA
Hepcidin H2H	0.38	0.04	NA	NA	NA
Hepcidin H2I	0.21	0.04	NA	NA	0.04

### In Vitro* Leucocyte Viability*

**Fig. 3:**
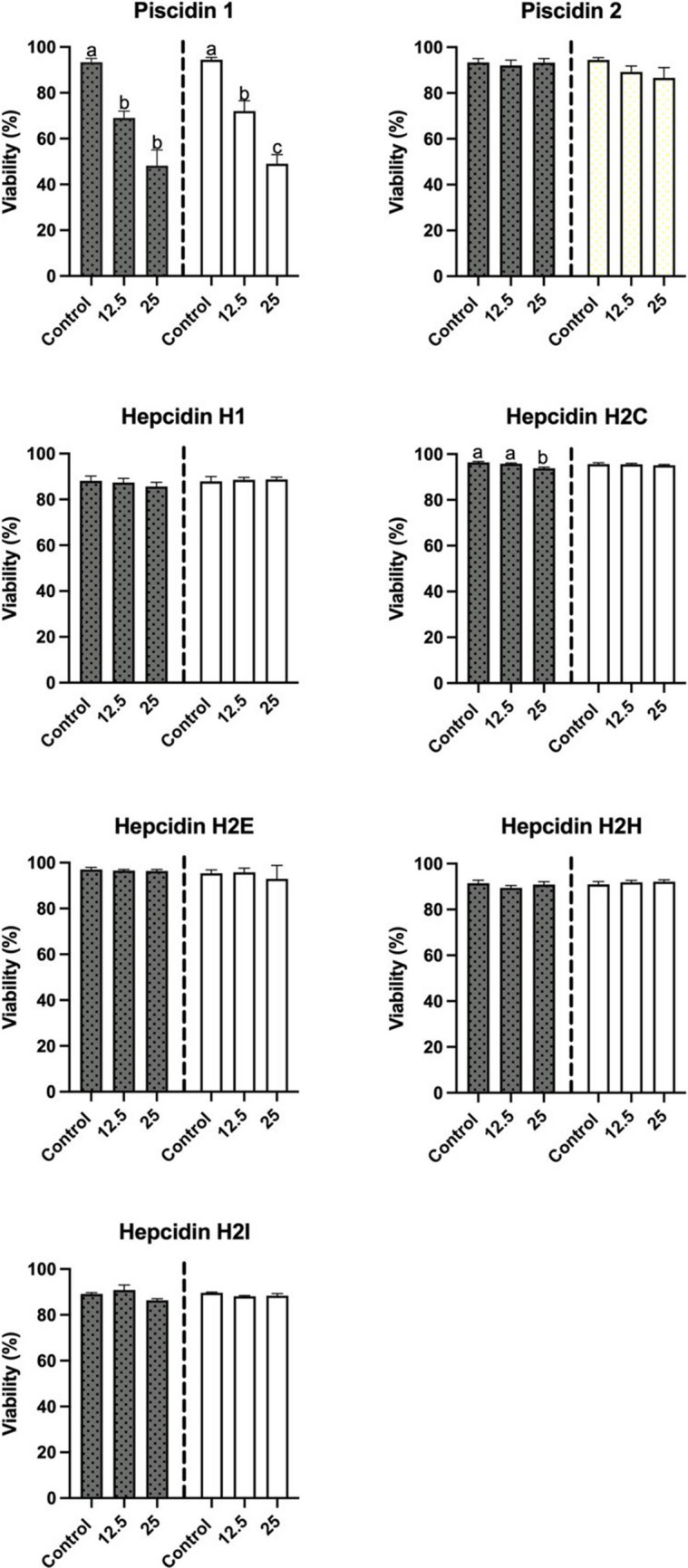
Viability of head-kidney leucocytes (expressed by the percentage of viable cells) after incubation for 0 h (grey) or 2 h (white) with different host defense peptides (HDPs) at concentrations of 0 µM, 12.5 µM and 25 µM. Data represents the mean ± standard error of the mean (SEM). Different letters indicate significant differences between experimental groups as determined by analysis of variance (ANOVA, *p* < 0.05)

Based on the results obtained from the incubation of seabream leucocytes with seven HDPs, it can be concluded that only two of them exhibited cytotoxic activity. Specifically, piscidin 1 was found to be the most cytotoxic, with its effects being dose dependent (*p* < 0.05). Furthermore, the results indicated that the decrease in leucocyte viability was similar when the cells were incubated for 0 h or 2 h with piscidin 1 (Fig. 3). On the other hand, hepcidin H2C was also found to have cytotoxic activity on seabream leucocytes; however, the decrease in leucocyte viability was only statistically significant (*p* < 0.05) when the cells were incubated for 0 h at 25 µM. No cytotoxicity was observed when the cells were incubated for 2 h with the same peptide. It is worth noting that no significant differences were observed in the cytotoxicity of leucocytes after incubation with the other HDPs.

### Immune Parameters of Leucocytes after Incubation with HDPs

**Fig. 4:**
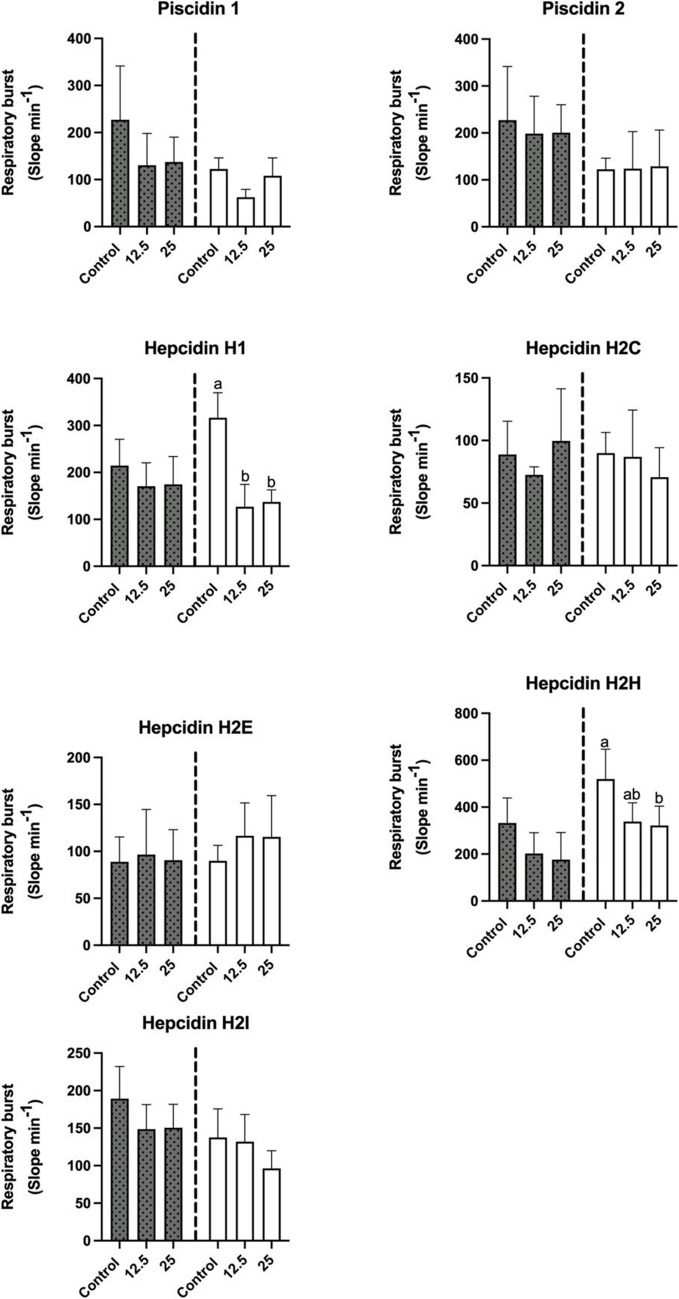
Respiratory burst of head-kidney leucocytes (expressed by the slope min^−1^) after incubation for 0 h (grey) or 2 h (white) with different host defense peptides (HDPs) at concentrations of 0 µM, 12.5 µM and 25 µM. Data represents the mean ± standard error of the mean (SEM). Different letters indicate significant differences between experimental groups as determined by analysis of variance (ANOVA, *p* < 0.05)

**Fig. 5:**
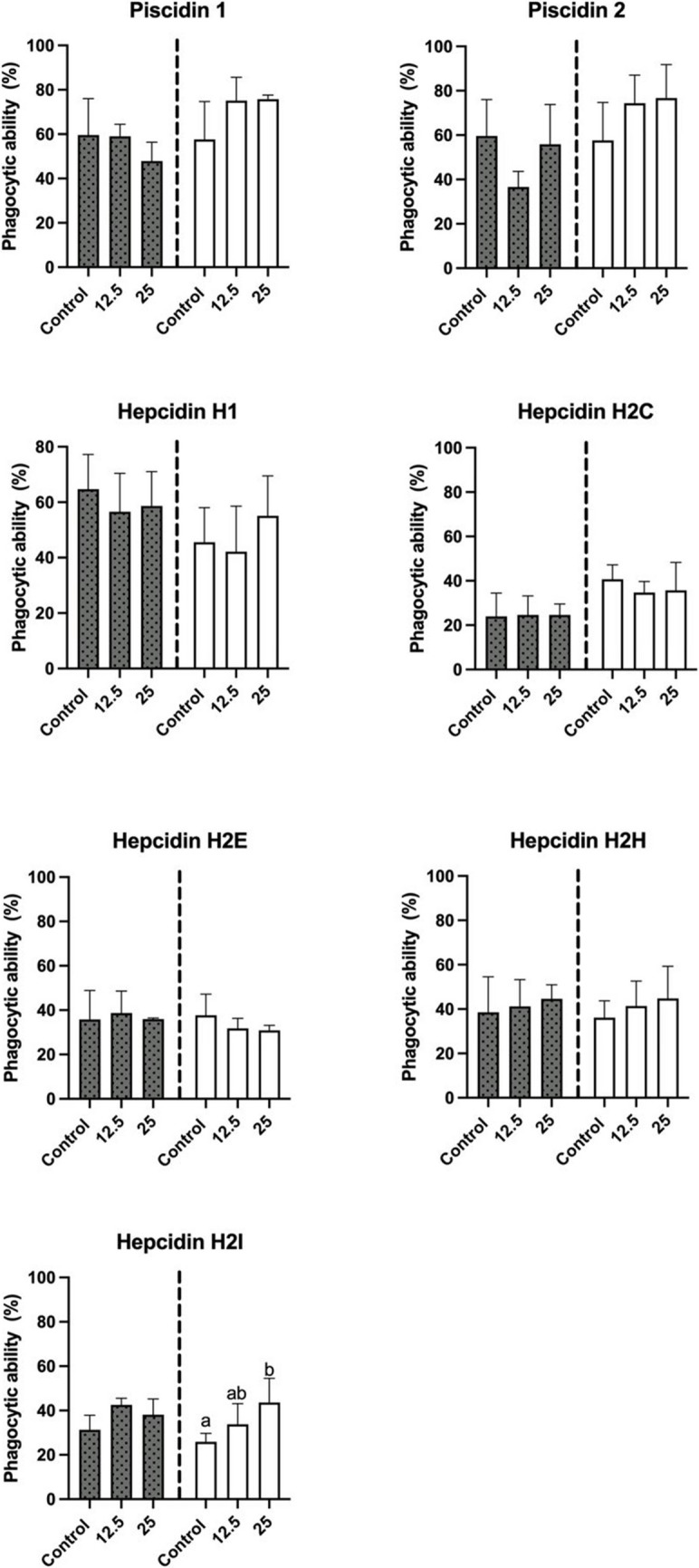
Phagocytic ability of head-kidney leucocytes (percentage of cells with ingested yeasts, %) after incubation for 0 h (grey) or 2 h (white) with different host defense peptides (HDPs) at concentrations of 0 µM, 12.5 µM and 25 µM. Data represents the mean ± standard error of the mean (SEM). Different letters indicate significant differences between experimental groups as determined by analysis of variance (ANOVA, *p* < 0.05)

**Fig. 6:**
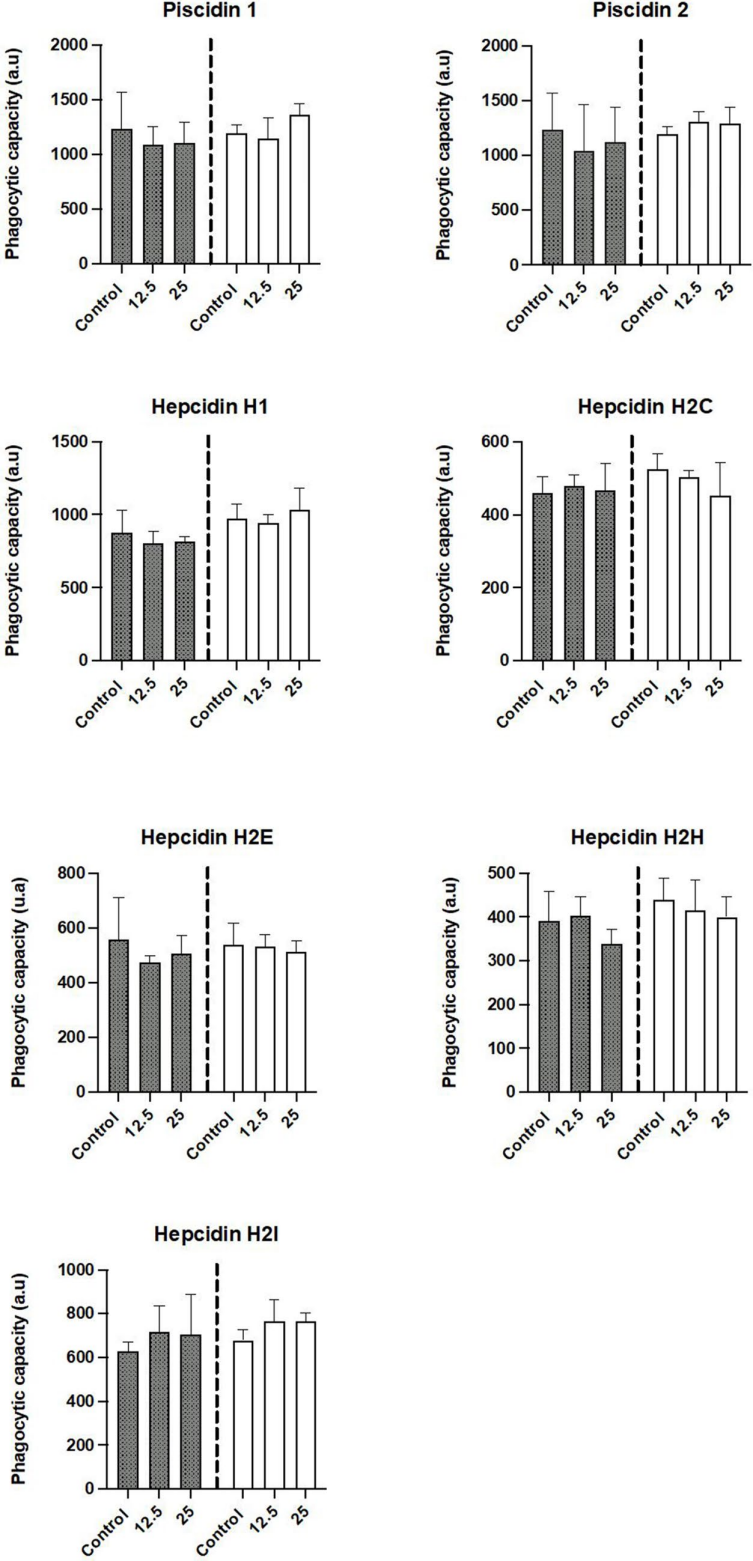
Phagocytic capacity of head-kidney leucocytes (expressed as arbitrary units, a.u) after incubation for 0 h (grey) or 2 h (white) with different host defense peptides (HDPs) at concentrations of 0 µM, 12.5 µM and 25 µM. Data represents the mean ± standard error of the mean (SEM). Different letters indicate significant differences between experimental groups as determined by analysis of variance (ANOVA, *p* < 0.05)

As for the influence of the peptides on respiratory burst, incubation of leucocytes for 0 h did not produce a decrease in this immune parameter (Fig. 4). Incubation of leucocytes for 2 h had different effects on respiratory burst, which were dependent on the peptide assayed. Significant decreases (*p* < 0.05), which were not dose-dependent, occurred in the respiratory burst when the leucocytes were incubated with hepcidin H1. The phagocytic ability of leucocytes incubated for 0 h with the different HDPs was not significantly affected (Fig. 5). However, when leucocytes were incubated for 2 h with hepcidin H2I at 25 µM there was a significant increase in this parameter (*p* < 0.05) (Fig. 4). Finally, incubation of leucocytes with the tested peptides for 0 h or 2 h did not produce any significant variation in their phagocytic capacity (Fig. 6).

### Correlation Analysis of Physicochemical Properties of HDPs and Leukocyte Viability and Immune Parameters after Incubation with HDPs

**Fig. 7:**
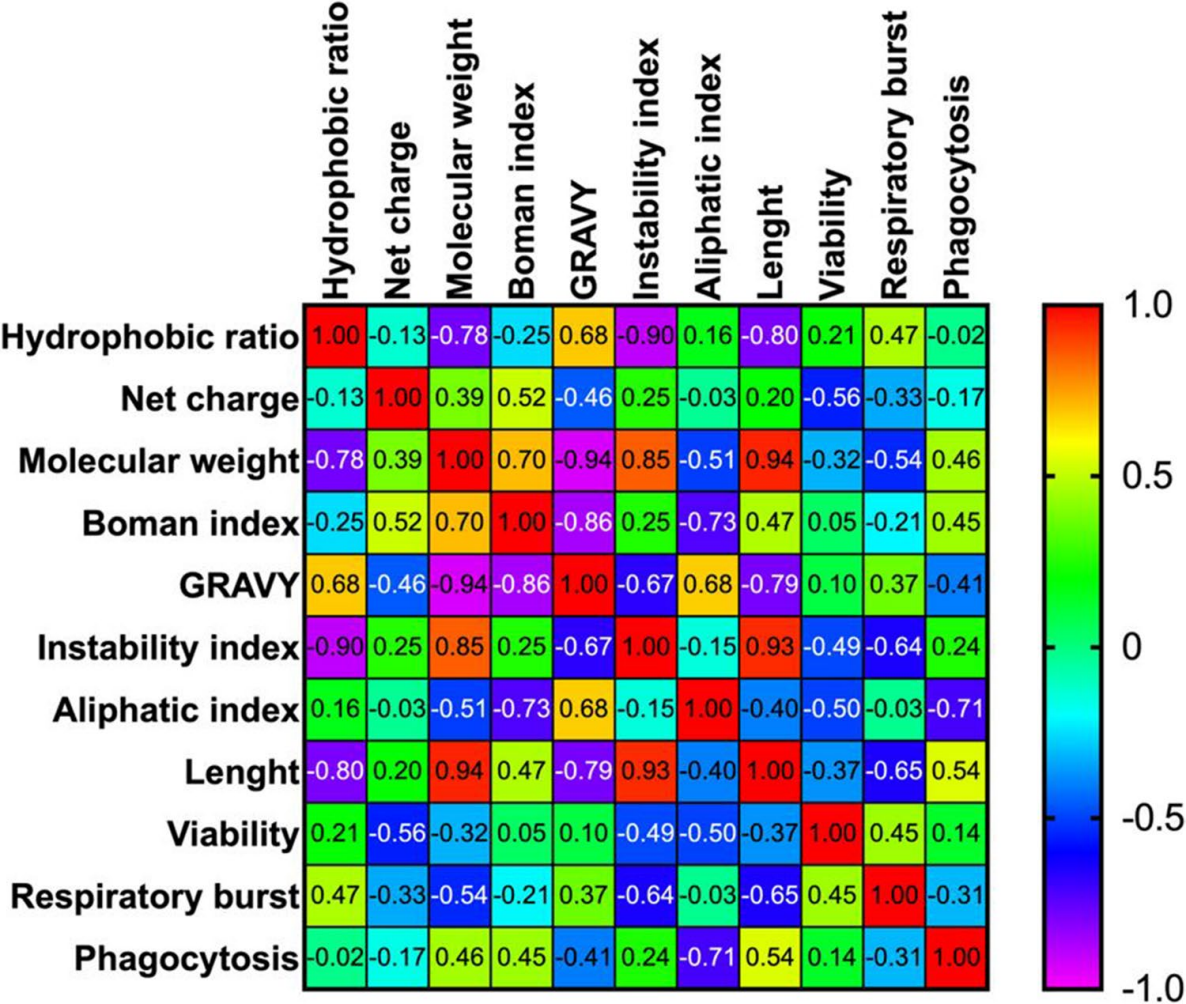
Correlation matrix of the relationship between physicochemical, viability and immune parameters. A positive correlation was considered when > 0.5

The correlation matrix analysis elucidated associations among the various parameters examined (Fig. 7). A positive correlation was observed between GRAVY and the hydrophobic ratio, GRAVY and aliphatic index, net charge and Boman index, molecular weight and Boman index/instability index/length, instability index and length, and length and phagocytosis. However, no positive correlation was detected between any parameter for leukocyte viability and respiratory burst.

### Gene Expression of Leucocytes after Incubation with HDPs

**Fig. 8:**
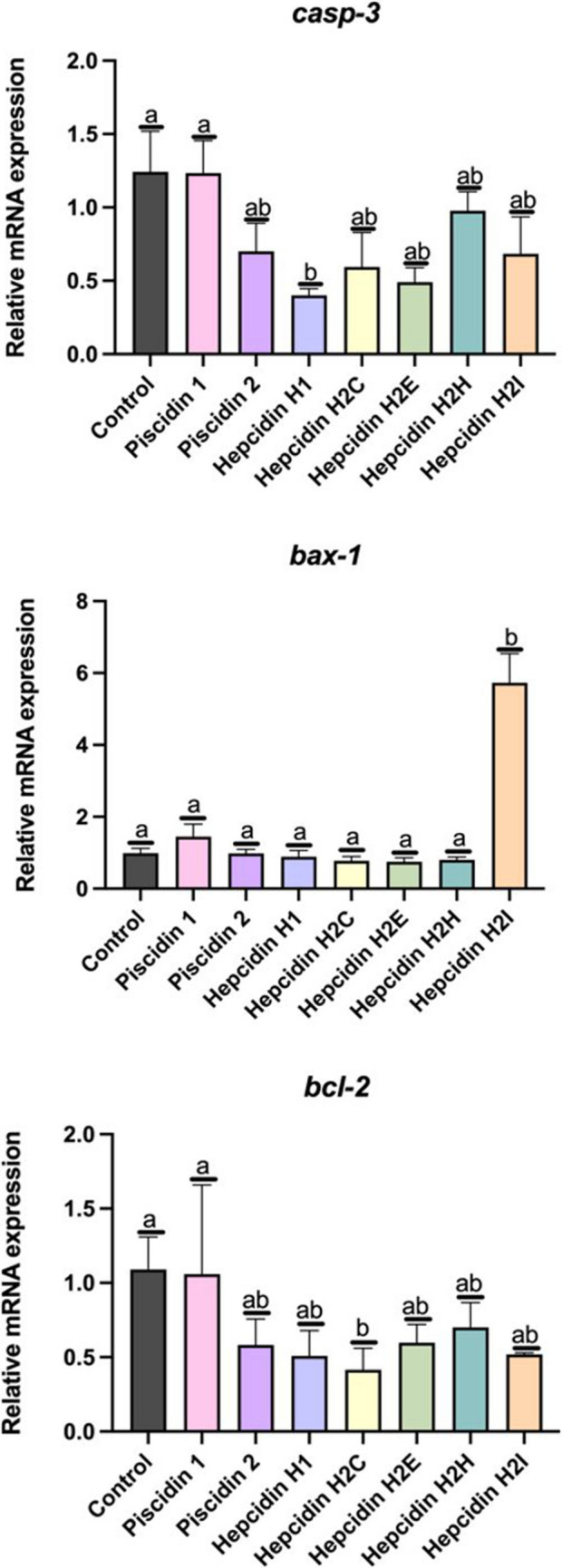
Relative mRNA expression level of: **a**) caspase 3 (*casp*−3), **b**) Bcl-2-associated protein X (*bax-1*), **c**) B-cell lymphoma 2 (*bcl-2*); in head kidney leucocytes from gilthead seabream incubated for 2 h with host defence peptides at 12.5 µM. Error bars in the columns indicate the standard error of the means. Different letters indicate significant differences between experimental groups as determined by analysis of variance (ANOVA, *p* < 0.05)

Variations in the expression of the three apoptosis-related genes in leucocytes incubated with HDPs for 2 h were also studied. When gilthead seabream leucocytes were incubated with piscidin, 1 or 2, the expression of these genes was not significantly altered (p > 0.05) (Fig. 7). However, when incubated with hepcidins, the results varied depending on the hepcidin used in the assays. Thus, *casp-3* expression was significantly decreased in leucocytes incubated with hepcidin H1. The expression of *bax-1* was significantly increased in leucocytes incubated with hepcidin H2I, whereas the expression of *bcl-2* was significantly decreased only in leucocytes incubated with hepcidin H2C (Fig. 8).

**Fig. 9:**
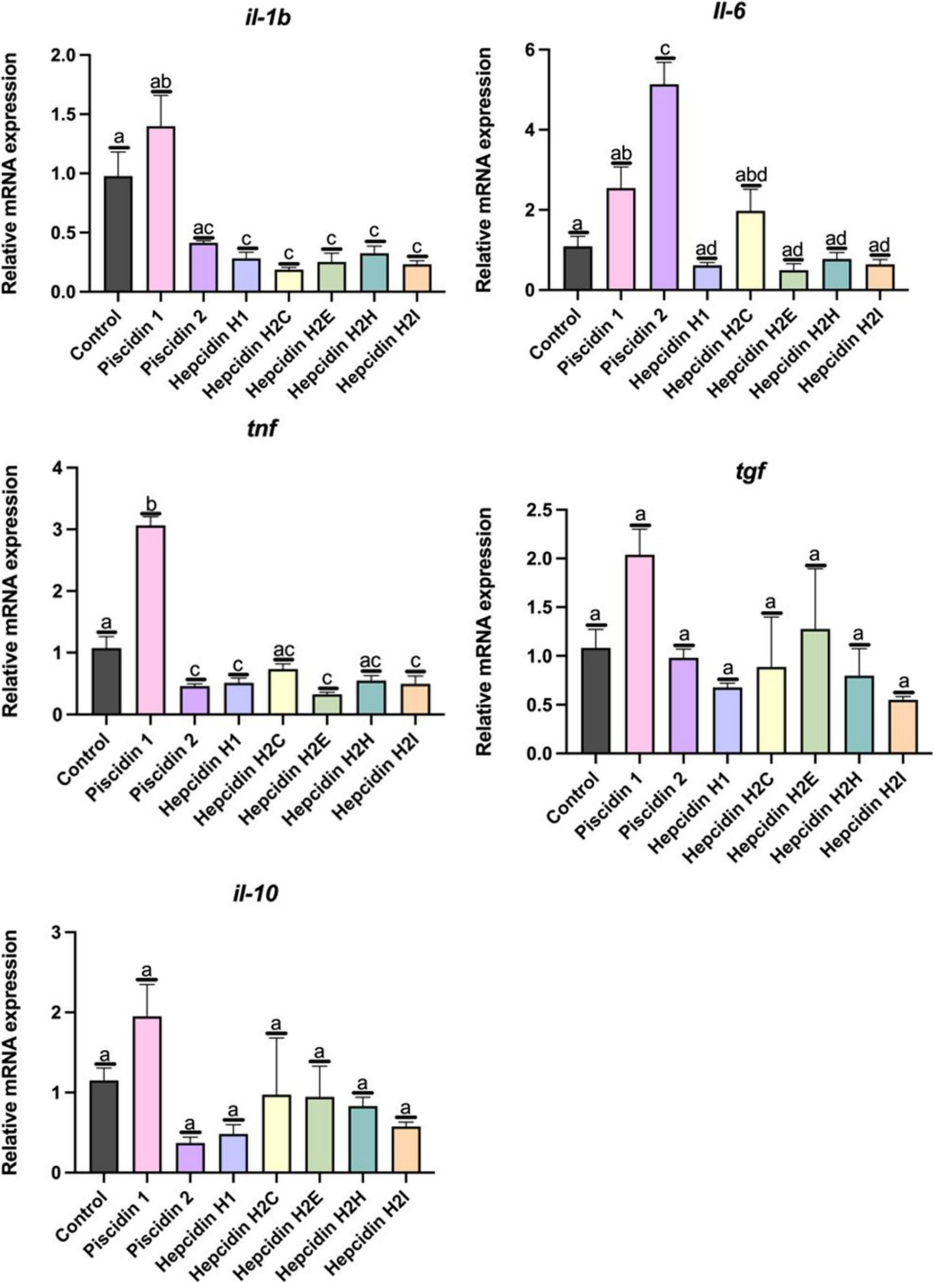
Relative mRNA expression level of: **a**) interleukin-1b (*il1-b)*, **b**) interleukin-6 (*il-6)*, **c**) tumor necrosis factor (*tnf-a)*, **d**) transforming growth factor *(tgf-β*) and **e**) interleukin-10 (*il10*); in gilthead seabream head kidney leucocytes incubated for 2 h with the host defense peptide at 12.5 µM. Error bars in columns denote the standard error of means. Different letters indicate significant differences between experimental groups determined by analysis of variance (ANOVA, *p* < 0.05)

Regarding the expression of the five genes involved in inflammation, *il-6* was significantly increased (*p* < 0.05), in leucocytes incubated with piscidin 2, with respect to control leucocytes (Fig. 8). *Tnf-α* expression was significantly increased in leucocytes incubated with piscidin 1 but decreased when incubated with piscidin 2). A significant decrease (*p* < 0.05) was observed, in the expression of *il1-β* in leucocytes incubated with any of the hepcidins tested (Fig. 9). The expression of *tnf-α* was significantly decreased (*p* < 0.05) with respect to the control in leucocytes incubated with hepcidin H1, H2E or H2I. The expression of *tgf-β* and *il-10* was not affected when leucocytes were incubated with any of the HDPs tested (Fig. 9).

**Fig. 10:**
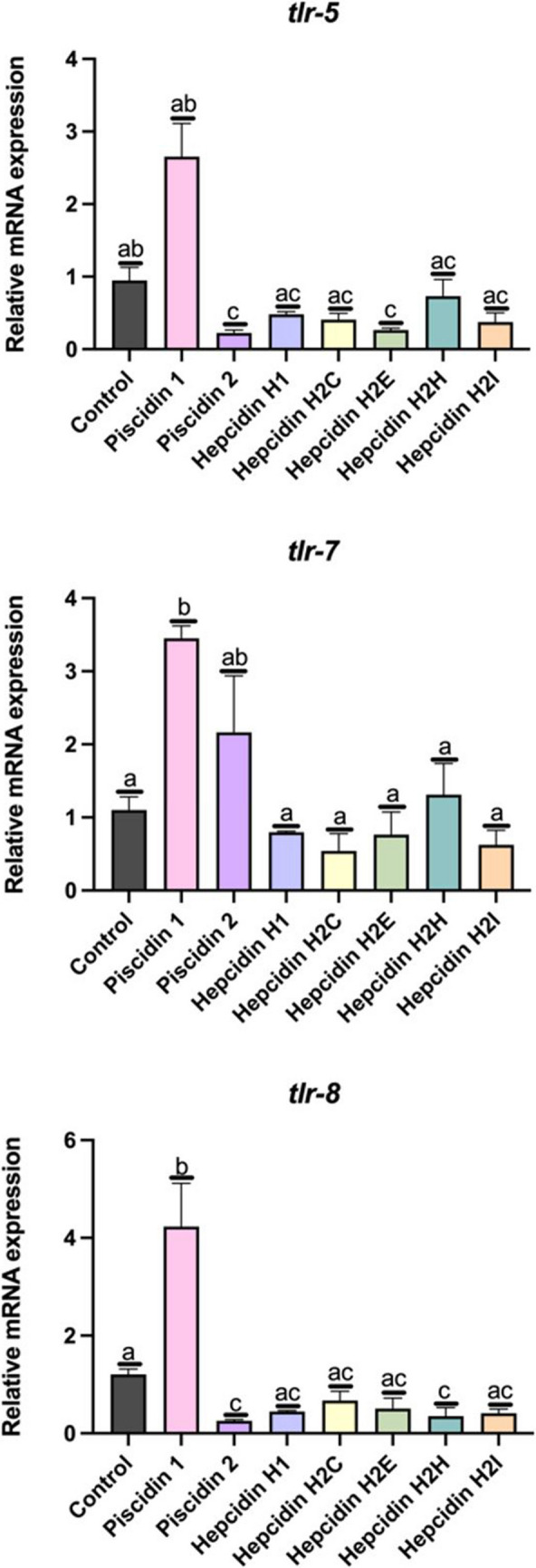
Relative mRNA expression of: **a**) toll-like receptor 5 *(tlr-5)*, **b**) toll-like receptor 7 (*tlr-7)* and **c**) toll-like receptor 8 (*tlr-8*); in gilthead seabream head kidney leucocytes incubated for 2 h with the host defense peptide at 12.5 µM. Error bars in columns denote the standard error of means. Different letters indicate significant differences between experimental groups determined by analysis of variance (ANOVA, *p* < 0.05)

Finally, the variation in expression of 3 toll-like receptors (TLR), *tlr-5, tlr-7* and *tlr-8*, was studied. Incubation of gilthead seabream leucocytes with piscidin 1 increased the expression of these two genes (*tlr-7* and *tlr-8*) in a statistically significant manner (*p* < 0.05) (Fig. 9). However, incubation of leucocytes with piscidin 2 significantly decreased the expression of *tlr-5* and *tlr-8*, relative to the expression of control leucocytes. The expression of the *tlr-5* decreased significantly (*p* < 0.05) when incubated with hepcidin H2E. The expression of *tlr-7* was not affected when leucocytes were incubated with hepcidins. The expression of *tlr-8* decreased (*p* < 0.05) in leucocytes incubated with hepcidin H2H (Fig. 10).

## Discussion

Based on research conducted over the past decade, it is widely accepted that a combination of computational and experimental approaches represents the most effective method for discovering novel HDPs and characterizing numerous biological activities. The use of in silico techniques for structural and functional characterization has proven to be beneficial, allowing researchers to identify that many marine AMPs are rich in cysteine residues (Sperstad et al. 2011; Wang et al. 2023). This study utilized in silico analysis to obtain some of the key characteristics of the selected HDPs. Subsequently, in vitro functional assays were developed to determine the biological effects of direct interactions between synthetic HDPs and gilthead seabream leucocytes, including cell viability and the main immune functions (respiratory burst and phagocytosis). Additionally, the effects of the HDPs on the modulation of genes related to apoptosis, inflammation, and toll-like receptors were also examined.

The results of in silico research demonstrate the extensive diversity of HDPs, as evidenced by various indices. This diversity reflects not only the distinctions between the two piscidins but also the variations observed among the five hepcidins analysed. Such disparities suggest that the interaction of HDPs with leucocytes will also exhibit considerable variation, thereby influencing the physiological effect they may produce. The integration of bioinformatics, machine learning algorithms, and advanced computational predictions has enabled researchers to efficiently and cost-effectively predict the activities of AMPs, leading to the identification of promising peptide candidates (Liscano et al. 2020). The in silico analysis reveals significant differences between the two studied piscidins in various indices. Previous studies on paralogous genes of gilthead seabream piscidins indicate that they encode different proteins based on their physicochemical properties. Moreover, the expression of basal and stimulated piscidin proteins, along with their putative genes, demonstrates distinct functions depending on the gene type, supporting the sub functionalization of duplicated genes (Serna-Duque et al. 2022b). Both piscidins also exhibit different effects on leucocyte viability and modulation of gene expression, which will be further discussed. Similarly, in silico work demonstrates significant differences between hepcidins in various indices. A previous study showed a positive correlation between hydrophobicity and GRAVY in antimicrobial peptides, whereas peptide length showed a positive correlation with immunomodulation (Trejos et al. 2023). These findings are consistent with the results of this study in which positive associations were observed between hydrophobicity and GRAVY, and peptide length and phagocytosis. These correlations may help to identify the physicochemical properties that most affect peptide functionality and improve the design of future peptides prior to the experimental stage.

It is crucial to discover novel immunomodulators to advance sustainable aquaculture practices, focusing on reducing dependence on therapeutics, enhancing disease management, ensuring environmental sustainability, and promoting costs reduction. Immunomodulators may improve disease management, decrease the reliance on antibiotics, boost vaccine efficiency, and improve the welfare of fish (Elumalai et al. 2023). Despite their potential, marine HDPs have been comparatively less studied than those from terrestrial environments, they possess qualities that make them appealing for enhancing the resilience of cultured aquatic organisms. Their broad-spectrum effectiveness, unique mechanisms of action, minimal harm to healthy cells, and exceptional stability are noteworthy attributes (Thomas and Antony 2024). However, one of the limitations of the use of HDPs is their possible cytotoxic activity on eukaryotic cells. Fortunately, one of the most noticeable features of marine AMPs is their low cytotoxicity (usually against mammalian cells) and lack of haemolytic properties which need to be explored before developing a possible therapeutic usage (Bae et al. 2016; Hansen et al. 2020; Yang et al. 2023). In this study, cytotoxicity was detected only in gilthead seabream leucocytes incubated for 0 h and 2 h with piscidin 1 (in a dose-dependent manner but not in a time dependent manner) or incubated for 0 h with the highest tested doses of hepcidin H2C. Our results on piscidin 1 agree with those of similar studies carried out with piscidin 1 from hybrid striped bass using different cell types (Lee et al. 2014, 2007; Kumar et al. 2016). However, three synthetic HDPs (NK-lysin, hepcidin and and dicentracin) derived from European sea bass (*Dicentrarchus labrax*) resulted to be non-toxic for European sea bass and gilthead seabream head-kidney leucocytes (Cervera et al. 2024). These results corroborate that factors such as peptide length, charge, hydrophobicity, and secondary structure can impact their interactions with eukaryotic cells and influence their cytotoxicity (Rai et al. 2016). According to the in silico results, piscidin 1 and hepcidin H2C had high net charge and high hydrophobicity and both parameters could be responsible for their cytotoxicity on seabream leucocytes. On the other hand, our cytotoxic results seem to suggest that the effects caused by piscidin 1 on leucocytes are not reversible, whereas those caused by hepcidin H2C appear to be reversible. However, further studies would be needed to determine why hepcidin H2C is cytotoxic to leucocytes immediately upon contact but not after 2 h of incubation. These new studies would resolve whether the leucocytes, in that time, can recover and repairing the damage caused by hepcidin H2C, which eliminates the cytotoxic effects by activate repair systems, or whether the cell metabolism itself can eliminate hepcidin H2C as a result, for example, of processes of cell transport, cell excretion or elimination by detoxification systems. An alternative explanation for the observed significant decrease in head-kidney leucocyte viability after exposure to Hepcidin H2C (25 μM) at 0 h, with no subsequent changes noted after 2 h, could be attributed to the rapid and potent initial impact of HDPs on cells, resulting in an immediate reduction in cell viability. At higher concentrations, hepcidin H2C may induce substantial cell damage or death instantaneously, and consequently, no further decrease in viability occurs over the subsequent 2 h. This suggests that the peptides rapidly target susceptible cells, rendering their impact minimal on the remaining population. HDPs may not show a quantifiable MIC50 (minimum inhibitory concentration to inhibit 50% of cytotoxicity) due to various reasons and one of them is that many HDPs act through rapid, membrane-disruptive mechanisms rather than targeting specific biochemical pathways like conventional antibiotics, causing an immediate and often all-or-nothing effect that complicates precise MIC50 determination. Additionally, HDPs exhibit broad-spectrum activity without a clear dose–response curve, as their effectiveness may depend on local concentrations and environmental conditions rather than a specific threshold. HDPs can also be quickly degraded by host enzymes or bind to host tissues, making it difficult to maintain a consistent inhibitory concentration. Furthermore, some microbes may develop low-level resistance or tolerance to HDPs, resulting in variable responses rather than a distinct concentration-dependent inhibitory effect (Lin et al. 2021).

Besides the peptides properties already mentioned, the cytotoxicity of AMPs can be attributed to several factors, including their interactions with cells membranes, leading to disruption and potential toxicity. AMPs often exert their antimicrobial activity by disrupting microbial cell membranes. However, this mechanism can have off-target effects on eukaryotic cells, leading to cytotoxicity. These off-target effects can result from interactions with intracellular components or signalling pathways within fish cells, as it has been described in mammals'cells (Wang et al. 2021). For this reason, the selectivity of HDPs towards microbial membranes versus fish cell membranes is crucial in determining their cytotoxic effects. By understanding these factors contributing to the cytotoxicity of HDPs, new research could work towards developing strategies to mitigate these effects and enhance the therapeutic potential of HDPs for practical applications in farmed fish. Thorough evaluation of HDPs through in vitro and in vivo studies can help assess their cytotoxicity profiles and select the most promising candidates for further development. However, it has to be considered that at present, even those HDPs causing cytotoxic could be used, for example, utilizing delivery systems like nanoparticles or liposomes to encapsulate them, an strategy that can help target their action specifically to microbial cells, reducing exposure to fish cells and minimizing cytotoxic effects (Wang et al. 2021; Ron-Doitch et al. 2016).

HDPs are generally known for their microbicidal activity exerted directly on the target microorganism, but they can also be endowed with potent immunomodulatory and receptor-mediated chemotactic activities, which together explain their broad antimicrobial activity (against bacteria, protozoa, fungi, and both enveloped and non-enveloped viruses), as well as the difficulty of selecting resistant mutants against them (Mahlapuu et al. 2016; Hancock et al. 2016). Some HDPs have been shown to stimulate the respiratory burst in mammalian cells, particularly in phagocytes. This activation can enhance the production of ROS, which are very important components of the immune response against invading microorganisms. It is important to note that the effects of HDPs on the respiratory burst can vary depending on the specific peptide, the type of immune cells involved, and the context of the immune response (Li et al. 2023a). However, little is known about the immunomodulatory potential of piscidins and hepcidins on fish leucocytes respiratory burst, phagocytosis, and modulation of their gene expression (Cervera et al. 2024).

The present study was also carried out with a leucocyte suspension, in which granulocytes and macrophages were the most abundant cell types. The results indicated that hepcidin H1 and H2H decreased the respiratory burst of leucocytes after 2 h of incubation. These results are contrary to what was observed in mudskipper (*Boleophthalmus pectinirostris)* and orange-spotted grouper *(Epinephelus coicodes*) where synthetic BpLEAP-2 and piscidins enhanced the respiratory burst of macrophages in HDKs-like (MO/MΦ) cell line (Chen et al. 2016; Huang et al. 2024). In silico analysis using BIOPEP-UWM database predicted that gilthead seabream piscidin 1 and all five hepcidins may have antioxidant properties. The present results may suggest that hepcidins H1 and H2H have a free radical scavenging ability which could be the reason for the detected negative impact of such HDPs on the respiratory burst of the leucocytes.

On the other hand, many HDPs enhance the phagocytic activity of immune cells, such as neutrophils and macrophages. HDPs may promote the recognition and engulfment of microbial invaders by binding to the pathogens or by facilitating opsonization, a process in which foreign particles are coated for recognition by phagocytic cells (Lai and Gallo 2009). Present results demonstrated that the effects of HDPs on leucocyte phagocytosis were negligible. Interestingly, only hepcidin H2I increased the phagocytic ability (but not the phagocytic capacity) of gilthead seabream leucocytes after 2 h of incubation. These results contrast with those in the HDP literature because, in general, both recombinant and synthetic HDPs were reported to enhance phagocytosis activity. For example, Japanese flounder HKLs incubated with recombinant hepcidin (rPoHep2) for 3 h enhanced the phagocytic activity (Li et al. 2023b). Similarly, HKLs leucocytes from rainbow trout (*Oncorhynchus mykiss*) incubated with three synthetics salmonid cathelicidin showed an increase in phagocytic uptake (D'Este et al. 2016). In mammals, synthetic eCATH-2 induced phagocytosis in murine RAW264.7 macrophages (ATCC-TIB-71) (Coorens et al. 2017). Given that hepcidin H2I was the only peptide that increased the phagocytic activity of HKLs, considered their biological properties from the in silico study, the anionic net charge and hydrophobicity of the peptide may influence cellular recognition during phagocytosis. However, new studies at the subcellular level would be needed to understand the interaction of hepcidin H2I with the cell membrane to grasp how it could affect phagocytosis. Nevertheless, perhaps its amino acid composition could affect its activity on phagocytosis. In this regard, some immunologically inert amino acids (L, G, I, P, Y, K) and phagocytosis-stimulating amino acids (L, Y, P, K) have been described for murine phagocytes (Belokrylov et al. 1992). New studies should be done to see if hepcidin H2I could act as opsonins, activate or suppress the activation of the classical pathway of the complement system, and activate the NLRP3 inflammasome, as it has been described for other HDPs (Prohászka et al. 1997).

To date, the mechanism by which different AMPs modulate immune response remains unknown (Pan et al. 2011). In this study, to shed some light on the field of immunomodulation by HDPs, the modulation caused of several genes on gilthead seabream leucocytes was studied. Three of the selected genes are related to apoptosis, a programmed cell death that is genetically regulated and controlled (Park et al. 2023). *Bax* and *Bcl-2* encoded apoptotic and anti-apoptotic proteins, respectively, and the ratio of those two proteins determines the occurrence of cell apoptosis (Maione et al. 2015). *Casp-3* is one of the major effectors of apoptosis, and its activation indicates irreversible cell apoptosis. Incubation of leucocytes with hepcidin H2I increased the expression of *bax-1*, potentially leading to the release of pro-apoptosis proteins that triggers a cascade of events involving caspase activation (Brentnall et al. 2013). In the present study, hepcidin H2I did not induce the expression of *casp-3* in the leucocytes, which may be due to the timeframe selected on the experiment. However, the expression of *casp-3* was down-regulated after incubation of leucocytes with hepcidin H1. These results agree with those obtained in an in vivo experiment where synthetic tilapia hepcidin (TH1-5) inhibited *casp-3* expression in mice (Huang et al. 2011). Interestingly, incubation of leucocytes with hepcidin H2C down-regulated the expression of *bcl-2*. New findings argue that the inhibition of BCL-2 proteins is a good alternative in the field of targeted cancer therapy (Vogler et al. 2011). These results encourage further studies to corroborate whether hepcidin H2C from gilthead seabream could be considered as a potential candidate for future anti-cancer research in humans. As already discussed, incubation of leucocytes with 12.5 µM piscidin 1 decreased their viability. However, piscidin 1 did not modulate significantly the expression of any of the apoptosis related genes, suggesting that trigger another type of cell death in HKLs, different to apoptosis, which should be explore in future studies. Overall*,* results reflects the complexity of the interactions between HDPs and the signalling pathways involved in the cell responses (Saxton et al. 2023).

The secretion of pro-inflammatory cytokines (IL-1b*,* IL-6 and TNF-a) initiates the activation of mechanisms that enhance the immune response (Deckers et al. 2023). Incubation of HKLs with piscidin 1 or 2 modulates the expression of proinflammatory cytokines, specifically *il-6* and *tnf-α*, respectively. IL-6, a pleiotropic cytokine, can activate the immune response of gilthead seabream to infectious agents. In addition, IL-6 can induce hepcidin expression (Ramakrishnan et al. 2018). In this study, hepcidins did not modulate *il-6* gene expression in HKLs, but are able to down-regulate the pro-inflammatory cytokines *il-1b* and *tnf-a.* TNF-a is crucial in the regulation of acute and chronic inflammation, and HKLs incubated with piscidin 1 had increased significantly the expression of this gene. Activation of *tnf-a* by this peptide may could induce cell death by necroptosis or trigger an inflammatory response by stimulating cytokine secretion (Sun and Kanwar 2015; Gonzalez Caldito 2023; Brenner et al. 2015)*.*

Finally, it is now known that one of the mechanisms by which HDPs modulate the immune response involves HDP-mediated TLR signalling (Lee et al. 2019). TLRs recognise pathogens through the detection of conservative pathogen-associated molecular patterns (Medzhitov 2001). Incubation of HKLs with piscidin 2 or hepcidin H2E showed down-regulation of *tlr-5* expression, which is mainly activated by flagellin. TLR-7 and TLR-8 are responsible for the recognition of guanosine (G)- and uridine (U)-rich viral single-stranded RNAs (sRNAs) (Heil et al. 2004). Incubation of HKLs with piscidin 2 or hepcidin H2H downregulated the expression of *tlr-8*. This could imply that piscidin 2 and hepcidin H2E and H2H down-regulate the expression of *tlrs* in HKLs to prevent excessive immune response and avoid negative impact due to prolonged activation. Incubation of HKLs with piscidin 1 increased *tlr-7/tlr-8* gene expression, which may induce the proinflammatory signalling pathway through TLR-7/TLR-8 activation related to single-strain RNA (ssRNA) in HKLs.

The present study examined two piscidins and five hepcidins derived from gilthead seabream. Further investigation into HDPs from a variety of fish species could provide more comprehensive insights. The experiments utilized isolated leukocytes in vitro, which may not fully capture the functionality of HDPs in vivo, thus necessitating validation in live fish to assess real-world applicability. Additionally, the experiments were conducted with short incubation periods (0 and 2 h), potentially overlooking the prolonged effects of HDPs on leukocytes, an area warranting further exploration. While the study analyzed several genes, a more extensive analysis could elucidate the broader impacts of HDPs on cellular processes. Finally, although the study identified various effects of HDPs on leukocytes, the application of omics techniques could facilitate an understanding of the underlying mechanisms of these effects.

In conclusion, this study highlights and underlines the intricate immunomodulatory effects of HDPs in seabream HKLs. Despite the limited direct impact of the studied HDPs on the tested leucocyte activities, the results demonstrate a clear involvement of HDPs in modulating the expression of key genes for immunity. The up or down-modulation of genes related to apoptosis, proinflammatory cytokines and Toll-like receptors underlines the complex and variable nature of the immunomodulatory actions of HDPs. Nevertheless, these findings highlight the fundamental role played by HDPs in the regulation of fish immune responses, contributing to our understanding of the intricate interplay between innate immunity and peptide-based defence mechanisms. It is crucial to further investigate the specific mechanisms underlying all these effects to exploit the therapeutic potential of HDPs in aquaculture and in many other fields of interest such as veterinary medicine, medicine, or cosmetics, among others.

## Data Availability

No datasets were generated or analysed during the current study.
